# Identification of Proteins and Genes Expressed by *Methylophaga thiooxydans* During Growth on Dimethylsulfide and Their Presence in Other Members of the Genus

**DOI:** 10.3389/fmicb.2019.01132

**Published:** 2019-05-29

**Authors:** Eileen Kröber, Hendrik Schäfer

**Affiliations:** School of Life Sciences, Gibbet Hill Campus, University of Warwick, Coventry, United Kingdom

**Keywords:** dimethylsulfide, methanethiol (MeSH), methylotrophy, RNAseq, proteomics, pangenomics

## Abstract

Dimethylsulfide is a volatile organic sulfur compound that provides the largest input of biogenic sulfur from the oceans to the atmosphere, and thence back to land, constituting an important link in the global sulfur cycle. Microorganisms degrading DMS affect fluxes of DMS in the environment, but the underlying metabolic pathways are still poorly understood. *Methylophaga thiooxydans* is a marine methylotrophic bacterium capable of growth on DMS as sole source of carbon and energy. Using proteomics and transcriptomics we identified genes expressed during growth on dimethylsulfide and methanol to refine our knowledge of the metabolic pathways that are involved in DMS and methanol degradation in this strain. Amongst the most highly expressed genes on DMS were the two methanethiol oxidases driving the oxidation of this reactive and toxic intermediate of DMS metabolism. Growth on DMS also increased expression of the enzymes of the tetrahydrofolate linked pathway of formaldehyde oxidation, in addition to the tetrahydromethanopterin linked pathway. Key enzymes of the inorganic sulfur oxidation pathway included flavocytochrome *c* sulfide dehydrogenase, sulfide quinone oxidoreductase, and persulfide dioxygenases. A *sulP* permease was also expressed during growth on DMS. Proteomics and transcriptomics also identified a number of highly expressed proteins and gene products whose function is currently not understood. As the identity of some enzymes of organic and inorganic sulfur metabolism previously detected in *Methylophaga* has not been characterized at the genetic level yet, highly expressed uncharacterized genes provide new targets for further biochemical and genetic analysis. A pan-genome analysis of six available *Methylophaga* genomes showed that only two of the six investigated strains, *M. thiooxydans* and *M. sulfidovorans* have the gene encoding methanethiol oxidase, suggesting that growth on methylated sulfur compounds of *M. aminisulfidivorans* is likely to involve different enzymes and metabolic intermediates. Hence, the pathways of DMS-utilization and subsequent C_1_ and sulfur oxidation are not conserved across *Methylophaga* isolates that degrade methylated sulfur compounds.

## Introduction

Dimethylsulfide is a volatile methylated sulfur compound that has been associated with the ‘smell of the sea’ (Stiefel, 1996) and which plays a crucial role in the global sulfur cycle (Lomans et al., 2002). The observation of significant concentrations of DMS in the marine boundary layer (Lovelock et al., 1972) led to the realization that marine emissions of DMS are driving transfer of sulfur between the marine and terrestrial environment via the atmosphere. In addition, its atmospheric breakdown products (mainly sulfate, sulfur dioxide, and methanesulfonic acid) are important precursors for secondary organic aerosols. These play a role in climate feedbacks by reflecting solar radiation back to space and serving as cloud condensation nuclei (CCN), which support the formation of clouds that reflect further sunlight and thus may contribute to climate regulation (Charlson et al., 1987; Simó, 2001; Carslaw et al., 2010; Lana et al., 2011).

The marine environment represents the largest source of DMS to the atmosphere (Watts, 2000) and the amount of DMS available for sea-to-air transfer depends on a number of microbial pathways that lead to production of DMS and its degradation in the surface ocean (Schäfer et al., 2010; Curson et al., 2011, 2018; Lidbury I. et al., 2016). Bacterial degradation of DMS is a major sink for DMS (Kiene and Bates, 1990) and a number of methylotrophic marine bacteria that are able to grow using DMS as a sole source of carbon and energy have been described previously, some of these belong to the genus *Methylophaga* (Janvier and Grimont, 1995). *Methylophaga* species able to grow on methylated sulfur compounds include the restricted facultative species *M. sulfidovorans* (De Zwart et al., 1997), *M. aminisulfidivorans* (Kim et al., 2007), and *M. thiooxydans* (Boden et al., 2010). It was shown that the latter produces tetrathionate as end-product of its sulfur metabolism (Boden et al., 2010). In addition to DMS, *M. thiooxydans* can grow on the one-carbon compounds methanol, dimethylamine (DMA), trimethylamine (TMA), as well as on fructose as sole carbon source (Boden et al., 2010). Boden et al. (2010) characterized the DMS-degradation pathway in *M. thiooxydans* based on enzyme assays and analysis of sulfur intermediates and end products and compared it to *M. thiooxydans* grown on methanol. Methanol dehydrogenase activity was detected in biomass grown on methanol and DMS; however, no enzyme activities were detected for degradation of DMS and methanethiol, an intermediate of DMS degradation, during growth on methanol (Boden et al., 2010). Furthermore, during growth on DMS, DMS monooxygenase (De Bont et al., 1981; Boden et al., 2010) activity was not detected, and the presence of a methyltransferase (Visscher and Taylor, 1993), which could be responsible for the initial step of DMS degradation, was suggested (Boden et al., 2010) although this ‘DMS-methyltransferase’ was not identified.

The aim of this study was to further characterize the metabolic pathways contributing to DMS degradation in *M. thiooxydans*. Proteomics and transcriptomics analyses were carried out with *M. thiooxydans* DMS010 in order to identify enzymes involved in DMS degradation in this model organism and potentially identify candidates of thus-far unidentified enzymes for further characterization.

## Materials and Methods

### Cultivation of *Methylophaga thiooxydans*

*Methylophaga thiooxydans* DMS010 was grown at 25°C in triplicate cultures using sterile marine ammonium mineral salts (MAMS) medium (Thompson et al., 1995), supplemented either with 1 mM methanol or 1 mM DMS as a sole carbon source and a 10% (v/v) inoculum. The cultures were monitored twice daily by measurement of optical density at 560 nm (OD560) in an UltrospecTM 3100 pro spectrophotometer (Amersham Biosciences, Corp., Piscataway, NJ, United States). Quantitative determination of DMS was as described previously (Lidbury I. et al., 2016). Cells were harvested at an OD560 of approximately 0.4 (exponential growth phase) by centrifugation at 13,000 × *g* for 30 min at 4°C. The supernatant was discarded, and cells were washed and resuspended in 50mM PIPES buffer (1,4-piperazinediethanesulfonic acid, pH 7.8) or TRIzol^®^ reagent (Life Technologies Corporation, Carlsbad, CA, United States) for proteomics or transcriptomics, respectively. Cells were then snap-frozen in liquid nitrogen and stored at -80°C until further processing.

### Proteomics

#### Protein Extraction and Quantification

For proteomics, proteins were extracted from triplicate *M. thiooxydans* DMS010 cell culture pellets resuspended in PIPES buffer. The homogeneous cell suspension was centrifuged at 9855 × *g* for 20 min at 4°C. The supernatant was discarded and the pellet was resuspended in 1 mL of an ice-cooled PIPES buffer with addition of 160 μg mL^-1^ benzamidine and 1 μg DNase was added. The PIPES buffer with 160 μg mL^-1^ benzamidine was prepared by mixing 0.39 g of benzamidine hydrochloride hydrate (98%) (Sigma-Aldrich) with 5 mL water. 0.2 mL of this solution was then mixed with 100 mL PIPES buffer and cooled on ice. Another 2 mL of this PIPES buffer (160 μg mL^-1^ benzamidine) was added and the cells were broken by three passages through a French pressure cell (American Instrument Corporation, Hartland, WI, United States) at 1000 psi. Cell debris were removed by centrifugation at 9855 × *g* for 20 min at 4°C. The supernatant was centrifuged again at 106,934 × *g* for 45 min at 4°C. After centrifugation the supernatant was transferred into 4.5 mL tubes (soluble protein fraction). The pellet was resuspended in 4.5 mL PIPES buffer (pH 7.8) and centrifuged again at 106,934 × *g* for 45 min at 4°C. Pellet resuspension and centrifugation was repeated, the supernatant was discarded and the pellet was resuspended in 500 μL PIPES buffer (without benzamidine).

Protein concentrations were determined using the Bradford protein assay (Bradford, 1976) and samples of the protein preparations were checked via sodium dodecyl sulfate polyacrylamide gel electrophoresis (SDS-PAGE) using a precast Mini-PROTEAN^®^ gel (Bio-Rad Laboratories, Inc.) and a Mini-PROTEAN^®^ Tetra cell (Bio-Rad Laboratories, Inc.). About 15 μg of protein was loaded onto the gel per sample and 20 μL of Precision Plus ProteinTM Standard (Dual Color, Bio-Rad Laboratories, Inc.). The gel was run at 200 V for about 35 to 40 min. Gels were stained overnight with InstantBlueTM protein stain (Sigma-Aldrich). SDS-PAGE analysis of soluble and membrane protein fractions showed reproducible protein profiles of replicates within each fraction type and carbon source but revealed different profiles of soluble and membrane fraction between carbon sources (Supplementary Figure S1).

#### Protein Identification and Data Analysis

The soluble protein fractions of *M. thiooxydans* DMS010 grown either on DMS or methanol (triplicates) were submitted for proteomic analysis to the Proteomics facility of the School of Life Sciences at the University of Warwick. The samples were subjected to a tryptic digest (Lidbury I.D.E.A. et al., 2016) followed by high-resolution mass spectrometry analysis using the Orbitrap Fusion Tribrid mass spectrometer (Thermo Fisher, Bremen, Germany). Peptide sequences obtained from MS/MS spectra were searched against *M. thiooxydans* DMS010 peptide sequences (database) using Mascot (Matrix Science, Inc., Boston, MA, United States) in order to identify proteins. Visualization and validation of the peptides was done with Scaffold (Proteome Software, Inc., Portland, ME, United States). Identified peptides and proteins were searched and placed into a pathway map using KEGG, Swiss-Prot, ExPASy, and UniProt. The mass spectrometry proteomics data have been deposited to the ProteomeXchange Consortium via the PRIDE partner repository with the dataset identifier PXD011992 and 10.6019/PXD011992^1^.

### Transcriptomics

#### RNA Extraction and Quantification

RNA was extracted from *M. thiooxydans* cell pellets resuspended in TRIzol^®^ reagent following the manufacturer’s instructions. Quantification of RNA was performed using the ND-1000 spectrophotometer (NanoDrop Technologies, Inc.). In addition, the concentration and purity of the RNA was also determined on a Bioanalyzer (Agilent 2100 Bioanalyzer, Agilent Technologies, Inc., United States) using an mRNA Nano chip (Agilent, Supplementary Figure S5).

#### rRNA Depletion

Depletion of rRNA was carried out using the Ribo-ZeroTM Magnetic kit for bacteria (Epicentre, Madison, WI, United States) following the manufacturer’s instructions. Enrichment of mRNA was checked on the Agilent 2100 Bioanalyzer using an mRNA Nano chip (Agilent).

#### Library Preparation and RNA Sequencing

Following rRNA depletion, samples were submitted to the Genomics facility of the School of Life Sciences at the University of Warwick for library preparation and RNA-sequencing (Illumina Hi-Seq 4000). The Genomics facility at the University of Warwick provided raw fastq reads.

#### RNA Sequencing Data Analysis

RNA sequencing reads were analyzed using Rockhopper (McClure et al., 2013). The reference-based transcriptome analysis was carried out by aligning the reads to a *M. thiooxydans* DMS010 reference genome which was constructed on the Rapid Annotation Server (RAST) using scaffolds available from the National Center for Biotechnology Information (NCBI) (ASM15635v1). Then data was normalized in Rockhopper in order to allow for data comparison between DMS-grown and methanol-grown *M. thiooxydans* DMS010 cultures. Afterward, transcripts were assembled, transcript boundaries were identified and transcript abundance was quantified. Finally, a test for differential gene expression was carried out in Rockhopper. Rockhopper analyses the data by aligning the reads to the *M. thiooxydans* DMS010 genome. Normalization between the triplicate experiments and different conditions (DMS/methanol) is done by upper quartile normalization. The transcripts are assembled and transcript boundaries are identified. Quantification of transcript abundance is done in RPKM (reads per kilobase per million mapped reads). RPKM is a common measure for counting gene expression and sums the number of reads for a gene and divides by the gene’s length and the total number of reads (McClure et al., 2013). Rockhopper reports the expression level, calculated from the triplicates, using RPKM. However, instead of dividing by the total number of reads, Rockhopper divides by the upper quartile of gene expression which has been suggested as a more robust normalization method (McClure et al., 2013). RNAseq data have been deposited in the NCBI Short Read Archive under Accession No. PRJNA509071.

A comparison between proteomics and transcriptomics was done by mapping the expression data of both approaches onto the *M. thiooxydans* genome using the CGView comparison tool using an in-house pipeline by Paul Stothard (Grant et al., 2012).

### Pan-Genome Analysis

#### Genome Data Acquisition

Six *Methylophaga* genomes (*M. thiooxydans* DMS0101, *M. sulfidovorans* DSM11578, *M. aminisulfidovorans*, *M. lonarensis*, *M. nitratireducenticrescens* strain JAM1, *M*. *frappieri* strain JAM7) available through the Integrated Microbial Genomes (IMG) database^2^ were used for comparative genome analysis (Markowitz et al., 2013). Accession numbers and additional genome characteristics are listed in Supplementary Table S1.

Pan-genome analysis including average amino acid identity (AAI) analysis, pan-genome tree construction and determination of core, dispensable genes and singletons (unique genes) was carried out using the Efficient Database framework for comparative Genome Analyses using BLAST score Ratios (EDGAR) platform (Blom et al., 2016).

In order to compare the genetic potential for dimethylsulfide degradation and sulfur oxidation within the available *Methylophaga* genomes, known protein sequences involved in dimethylsulfide degradation and sulfur oxidation pathways were used as query sequences through the BLAST (blastp) program (Altschul et al., 1990) available within the Rapid Annotation using Subsystem Technology (RAST) server (Aziz et al., 2008). The list of protein queries used is given in Supplementary Table S2.

## Results

### Known Genes in *Methylophaga thiooxydans* Genome With Potential Roles in Methanol and DMS Based Methylotrophy and Sulfur Oxidation

A summary of the genetic potential of *M. thiooxydans* with particular relevance to methanol and DMS metabolism is given below.

#### Methanol Oxidation

The draft genome sequence of *M. thiooxydans* contains four genes encoding methanol dehydrogenases (MDH) including a Calcium-dependent methanol dehydrogenase (MxaFI, locus tag MDMS009_1502 encoding the alpha subunit, and MDMS009_1410, beta subunit) and three XoxF-type methanol dehydrogenases (MDMS009_1767, _2058, and _2642) which are homologs of the MDH enzymes shown to require rare earth elements as co-factors (Keltjens et al., 2014). Genes *pqqBCDE* (encoded by MDMS009_2015, MDMS009_1956, MDMS009_1782, MDMS009_1752, respectively) involved in the synthesis of pyrroloquinoline quinone (PQQ), the co-factor of MDH, are co-located in a four gene cluster in the vicinity of two of the XoxF encoding genes (MDMS009_1767 and MDMS009_2058).

#### Formaldehyde Metabolism

Formaldehyde generated through primary metabolism of C_1_ substrates such as methanol, DMS and methanethiol can be conjugated to tetrahydromethanopterin (H_4_MPT) by formaldehyde activating enzyme (Fae) for which two coding sequences are found (MDMS009_73 and MDMS009_595). There is a complete H_4_MPT pathway for formaldehyde oxidation, comprising methylene-H_4_MPT dehydrogenase (*mtdB*, MDMS009_1418), methenyl-H_4_MPT-cyclohydrolase (*mch*, MDMS009_650), formylmethanofuran-H_4_MPT-N-formyltransferase (*fhcD*, MDMS009_1442) and formyltransferase/hydrolase complex (*fhcA/fhcB/fhcC*, MDMS009_1429/MDMS009_1334/MDMS009_1520), which releases formate. Similarly, formate can be produced from C_1_ groups fed into a tetrahydrofolate (H_4_F) linked C_1_-oxidation pathway comprising methylene-H_4_F reductase (*metF*, MDMS009_515), a bifunctional methylene-H_4_F-dehydrogenase/cyclohydrolase (*folD*, MDMS009_2980) which leads to production of formyl-H_4_F which can then be degraded to formate either by formyl-H_4_F-deformylase (*purU*, MDMS009_888) or the formate-H_4_F ligase (*fhs*, MDMS009_1028) which couples the oxidation of formyl-H_4_F to the production of ATP. The latter can also feed formate into the H_4_F pathway to reduce formate for assimilatory purposes.

Finally, subunits of a formate dehydrogenase which can oxidize formate to CO_2_ are encoded by MDMS009_2131, MDMS009_2239, MDMS009_2257, and MDMS009_1738.

*Methylophaga thiooxydans* has a ribulose monophosphate cycle (Entner–Doudoroff variant) for assimilation of formaldehyde including the enzymes 3-hexulose-6-phosphate synthase (*hxlA*, MDMS009_509), 6-phospho-3-hexuloisomerase (*hxlB*, MDMS009_475), fructose-bisphosphate aldolase (*fba*, MDMS009_1331), fructose-bisphosphatase (*fbp*_1, MDMS009_1532), and transketolase (*tkt*, MDMS009_866).

#### Methanethiol and Sulfide Oxidation

*Methylophaga* has two genes encoding methanethiol oxidase (MDMS009_211 and MDMS009_768) contained in two distinct genomic regions which also encode a number of other genes involved in sulfur metabolism referred to hereafter as mto-clusters 1 and 2, respectively (Supplementary Figure S2). The gene order in both clusters is identical, with the major difference being that the mto-cluster1 contains two additional genes at the end. Functions encoded in both clusters include (i) the methanethiol oxidase, (ii) a fusion of SCO1 with MauG-type cytochrome *c* peroxidase, (iii) a SCO1/SenC/PrrC domain protein predicted to be involved in cytochrome maturation (identified by conserved domain search as a TlpA-like_family putative metallochaperone), (iv) homologs of persulfide dioxygenase (PDO) annotated as hydroxyacylglutathione hydrolases, (v) FAD-dependent pyridine nucleotide-disulfide oxidoreductases (Sulfide quinone reductase B, SqrB), and (vi) a *sulP* gene encoding a sulfate/sulfite permease. In addition, mto-cluster 1 contains an adenylylsulfate kinase and a gene annotated as a ‘methylated-DNA-protein-cysteine methyltransferase’ in the same orientation, however to which extent the genes in the mto-clusters are co-regulated from one or more promoters is unclear.

In mto-cluster 2, the predicted methanethiol oxidase encoded by MDMS009_768 (465 amino acids [aa]) has a predicted signal peptide, but the mto-cluster 1 ortholog MDMS009_211 (427 aa) does not, prompting the question whether the former is periplasmic and the latter cytoplasmic. The region upstream of the CDS of MDMS009_211 has two alternative start codons, one of which would extend the encoded enzyme by 40 amino acids and result in a pre-MTO with a predicted signal peptide (SignalP 5.0 likelihood 98.2%, compared to 0.2% with previous start site, Supplementary Figure S3) as well as a more A/G rich ribosome binding site. In addition, for both mto-clusters the downstream *sco1/mauG* genes encoding proteins thought to be involved in MTO maturation are predicted to have a signal peptide. Together, these observations suggest that both MTO are periplasmic and are initially pre-proteins that are exported by the Sec pathway, where they are subsequently processed by the SCO1/MauG fusion proteins. The genes MDMS009_140 and MDMS009_894, immediately downstream of the SCO1/MauG genes, encoding SCO1/SenC/PrrC domain proteins are also expected to be periplasmic if an alternative start site of MDMS009_894 is taken into account (Supplementary Figure S4), which would result in products of similar length for these two proteins (168 and 164 aa, respectively), both with strong likelihood of containing a signal peptide (>98% likelihood).

Sulfide produced from MT oxidation can be oxidized by a flavocytochrome *c* sulfide dehydrogenase (*fccB*, MDMS009_2035; *fccA* MDMS009_753); genes encoding sulfide-quinone oxidoreductases include an *sqrA* MDMS009_1966 (based on homology to *Aquifex aeolicus sqrA*) as well as two *sqrB* (MDMS009_274, MDMS009_886). The location of these SQRs may be cytoplasmic or periplasmic, the confidence values for predicted signal peptide sequences are variable, 12% for MDMS009_274, 27% for MDMS009_1966, 55% for MDMS009_886. Candidate genes encoding persulfide dioxygenases include MDMS009_284 and MDMS009_765, both without signal peptides, suggesting these to be located in the cytoplasm. Permeases for sulfate or sulfite compounds are encoded by MDMS009_261 and MDMS009_921.

The genome of *M. thiooxydans* encodes some functions of the thiosulfate oxidation pathway including *soxCDHYZ*, as well as two genes encoding *soxYZ* fusions (MDMS009_1377 and 1373) but genes encoding SoxAB are missing suggesting this pathway cannot be used for oxidation of thiosulfate. Previous work suggested oxidation of thiosulfate to tetrathionate to occur, but the genome does not contain genes encoding any of the previously characterized thiosulfate to tetrathionate oxidizing enzymes, including an *Allochromatium vinosum* type TsdA thiosulfate dehydrogenase (Denkmann et al., 2012), a *Shewanella oneidensis* type octaheme tetrathionate reductase of Atkinson et al. (2007) or a homolog of DoxDA as shown in *Acidianus* or *Acidithiobacillus* species (Wang et al., 2016).

### The Differential Proteome of *Methylophaga thiooxydans* DMS010 From DMS and Methanol Grown Cultures

**FIGURE 1:**
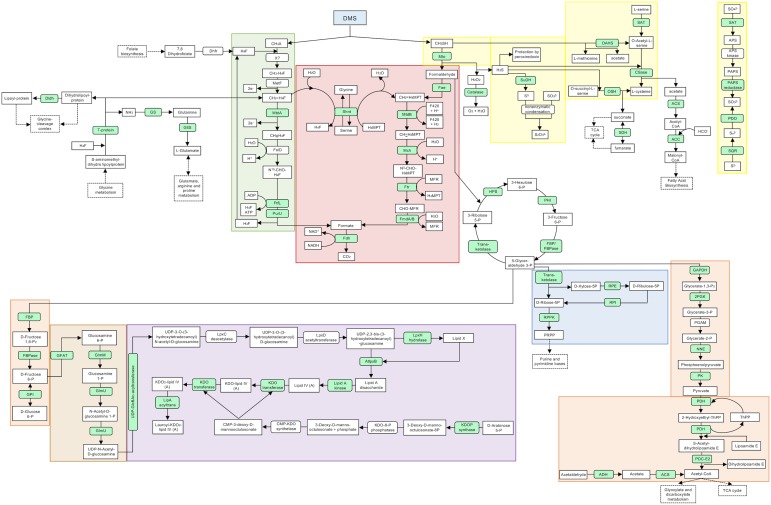
Proposed pathways and reactions in *Methylophaga thiooxydans* DMS010 grown on DMS. Metabolic map showing selected pathways of *M. thiooxydans* DMS010. Metabolic pathways, both predicted from genome and proteomic analysis are presented in a simplified bacterial cell. Proteins detected in the proteomic analysis are indicated in light green and those not detected are indicated in white. Main metabolites are shown in square boxes and enzymes are shown in oval boxes. Some KEGG categories are indicated by color; green, one carbon pool by folate; blue, pentose phosphate path-ways (parts); orange, glycolysis; beige/brown, amino sugar and nucleotide sugar metabolism; purple, lipopolysaccharide biosynthesis; red, methane metabolism and yellow, sulfur metabolism. A solid arrow stands for an enzymatic reaction and a dashed arrow stands for multistep pathway. Abbreviations are explained in more detail in Supplementary Table S4; X? – uncharacterized DMS methyltransferase.

Soluble and membrane protein fractions of *M. thiooxydans* DMS010 prepared for the proteomics analysis had concentrations ranging from 0.22 to >1 mg mL^-1^ (Supplementary Table S3). All six soluble protein fractions were submitted for proteomic analysis. Identified peptides and proteins (minimum of 99% protein identity) were searched and placed into a pathway map using KEGG, Swiss-Prot, ExPASy, and UniProt. A proposed pathway map of *M. thiooxydans* DMS010 grown with DMS is presented in Figure 1. A list of the corresponding proteins, genes, locus tag, and ratios of expression levels of the proteins expressed during growth on DMS or methanol can be found in Supplementary Table S4. Additional hypothetical proteins can be found in Supplementary Table S5.

Overall, up to 662 proteins (aggregate across triplicate samples) were identified in *M. thiooxydans* DMS010 when grown on methanol, from which up to 80 proteins were up-regulated (at least twofold) compared to *M. thiooxydans* DMS010 growing on DMS as a carbon source. Up to 813 proteins were identified in *M. thiooxydans* DMS010 grown on DMS, of which up to 177 were up-regulated compared to growth on methanol. Proteins highly up-regulated in *M. thiooxydans* DMS010 growing in the presence of DMS were the methanethiol oxidases, of which *M. thiooxydans* encodes two (on average 7- and 18.67-fold, respectively, higher compared to methanol grown *M*. *thiooxydans* DMS010; Supplementary Table S4) in line with previous reports of methanethiol being a metabolic intermediate of its DMS metabolism (Schäfer, 2007; Boden et al., 2010).

Degradation of methanethiol via methanethiol oxidase (Mto – Figure 1) results in formation of formaldehyde, hydrogen sulfide (H_2_S) and hydrogen peroxide (H_2_O_2_). H_2_O_2_ is toxic (Clifford and Repine, 1982; Thomas et al., 1994) and the catalase that decomposes it into water and oxygen was 14.67-fold up-regulated during growth on DMS (Figure 1). Similarly, peroxiredoxin was also up-regulated (3.75-fold) during growth on DMS, likely contributing to degradation of hydrogen peroxide or organic peroxides produced by H_2_O_2_ stress in the cell (Rhee, 2016).

The formaldehyde produced by methanethiol oxidase can either be degraded to CO_2_ via formate or be assimilated into biomass via the RuMP cycle. Several enzymes of the RuMP cycle were detected, such as 3-hexulose-6-phosphate synthase (MDMS009_509), 6-phospho-3-hexuloisomerase (MDMS009_475), transketolase (MDMS009_866), fructose-bisphosphate aldolase (MDMS009_1331) and fructose-bisphosphatase (MDMS009_1532). Formaldehyde degradation to formate and CO_2_ appeared to occur via both the tetrahydromethanopterin-linked pathway and the tetrahydrofolate-linked pathway (Supplementary Table S4). The expression of most enzymes of these pathways showed only minor variation in expression levels between growth on methanol and DMS, although methylenetetrahydrofolate dehydrogenase (MtdA) was 5.30-fold up-regulated on DMS potentially indicating that one of the methyl groups of DMS may be oxidized via this pathway after its transfer onto tetrahydrofolate (H_4_F) (Figure 1). In addition, the formate—tetrahydrofolate ligase (2.20-fold up-regulated) and the formyltetrahydrofolate deformylase of the tetrahydrofolate pathway were detected (Figure 1 and Supplementary Table S4). The expression of formate dehydrogenase was also shown (2.17-fold upregulated), which degrades the end-product of the tetrahydromethanopterin and tetrahydrofolate pathways, formate, to CO_2_ (Figure 1). In addition, enzymes related to the glycolysis pathway such as the glucose 6-phosphate isomerase (1.66-fold) that catalyzes the conversion of D-glucose 6-phosphate to D-fructose 6-phosphate, the phosphopyruvate hydratase (3.44-fold) or enzymes involved in the conversion of glyceraldehyde-3-phosphate to pyruvate were found upregulated. The enzymes dihydrolipoamide dehydrogenase (7.67-fold) and dihydrolipoamide *S*-acetyltransferase (7.50-fold, E2 component of the pyruvate dehydrogenase) were also highly upregulated. Furthermore, proteins involved in the metabolism of amino and nucleotide sugars, and lipopolysaccharide biosynthesis were also detected. The oxidation of the sulfur of DMS is of major importance as an intermediate in the sulfur cycle. The initial step of DMS degradation and the subsequent oxidation of the sulfur are summarized in Figure 1, 2. An enzyme of sulfide oxidation detected in the proteome of DMS-grown cells included sulfide dehydrogenase (MDMS009_2035; encoding FccB), which was upregulated 1.67-fold.

**FIGURE 2:**
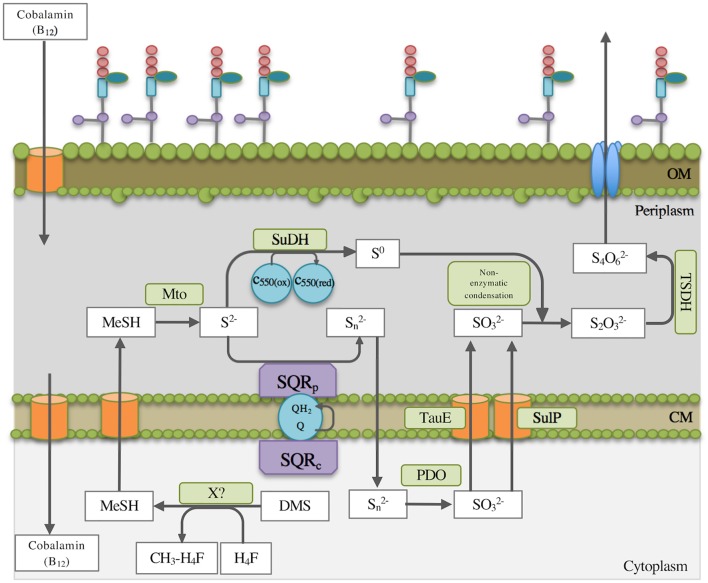
Simplified schematic overview of the proposed DMS degradation and subsequent route of sulfur oxidation in *Methylophaga thiooxydans* DMS010. Boden et al. (2010) suggested that DMS is initially degraded by a suggested cobalamin-dependent and as yet unidentified methyltransferase in the cytoplasm. Methanethiol (MT) would need to transfer to the periplasm where the methanethiol oxidase (Mto) degrades MT further as described in the text. The suggested steps of the sulfur oxidation pathway according to enzyme essays, proteomics and transcriptomics analyses are indicated. MT, methanethiol; X?, unidentified putative methyltransferase; H_4_F, tetrahydrofolate; CM, cytoplasmic membrane; OM, outer membrane; Mto, methanethiol oxidase; SuDH, sulfide dehydrogenase (flavocytochrome); SQR_p_, periplasmatic sulfide:quinone reductase; SQR_c_, cytoplasmatic sulfide:quinone reductase; Q, quinone, QH_2_, quinol; SulP, sulfate permease; TauE, sulfite exporter; PDO, persulfide dioxygenase; TSDH, thiosulfate dehydrogenase; C_550_, cytochrome 550 (located in the cytoplasmic membrane).

### The Differential Transcriptome of *Methylophaga thiooxydans* DMS010 From DMS and Methanol Grown Cultures

RNA extraction and rRNA depletion led to successful mRNA enrichment. Results of the RNA analysis using the Bioanalyzer can be found in Supplementary Figure S5. Concentrations of mRNA preparations from *M. thiooxydans* grown on methanol ranged from 14.5 to 26.98 pg/μL and for *M. thiooxydans* grown on DMS from 85.7 to 172.0 pg/μL. These mRNA preparations were used for sequencing library preparation and high-throughput sequencing of cDNA (from mRNA). Overall, between 82 and 89% of the transcriptomic reads of the triplicate DMS and methanol treatments were successfully aligned to the *M. thiooxydans* DMS010 genome. Between 23 and 27% aligned to unannotated regions. 1228 5′ UTRs and 1309 3′ UTRs (untranslated regions) were detected and 176,974 RNAs were predicted. From these RNA transcripts an overall 3,078 RNAs coding for proteins were predicted. Of those protein-coding transcripts, 280 were highly up-regulated (2.5- to 469-fold) and another 1,340 protein-coding transcripts were up-regulated at least twofold during growth on DMS compared to methanol. When *M. thiooxydans* was grown on methanol, 230 protein-coding transcripts were highly up-regulated (2.5- to 52-fold). Transcripts coding for 936 hypothetical proteins were detected, of which 133 were highly up-regulated (at least 2.5-fold) during growth on DMS. In total, 115 operons were detected by Rockhopper during the transcriptome analysis. Supplementary Table S6 summarizes the main results of the transcriptomic experiment by indicating the protein-encoding genes with locus tags and their expression level in *M. thiooxydans* when grown either with methanol or DMS. Highly up-regulated transcripts in the DMS treatment compared to methanol treatment were those of the genes encoding for methanethiol oxidase (MDMS009_211, MDMS009_768) and catalase/peroxidase (MDMS009_1525, MDMS009_2469), dihydrolipoamide dehydrogenase (MDMS009_809, a component of the pyruvate, α-ketoglutarate, and branched-chain amino acid-dehydrogenase complexes and the glycine cleavage system), several hypothetical proteins, proteins involved in coenzyme PQQ synthesis (*pqqE*, *pqqD*, and *pqqB*), a membrane-bound protein (MDMS009_906) potentially involved in long chain fatty acid transport (porin), a fatty acid desaturase (MDMS009_226) and several others (Supplementary Table S6). Highly up-regulated transcripts in the methanol treatment compared to the DMS treatment included proteins involved in siderophore (high-affinity iron-chelating compounds) biosynthesis and methanol dehydrogenase proteins (Supplementary Table S6).

### Comparison of Proteomics and Transcriptomics Data

A comparison of proteomics and transcriptomics data based on mapping the expression data of both approaches onto the *M. thiooxydans* genome using the CGView comparison tool in-house pipeline (Grant et al., 2012) is shown in Supplementary Figure S6. Overall, there was good agreement between the two approaches, whereby the transcriptome data showed additional detail, which is not surprising due to high-throughput sequencing providing more in-depth analysis and higher coverage (Hegde et al., 2003; Trapp et al., 2017) and the omission of the membrane fraction for proteomics analysis, for instance. The analysis against the genome assembly identified the activation of specific regions of the genome strongly supporting coordinate gene expression depending on the growth substrate (Supplementary Figure S6).

### Comparative Genome Analysis of *Methylophaga* Isolates

**FIGURE 3:**
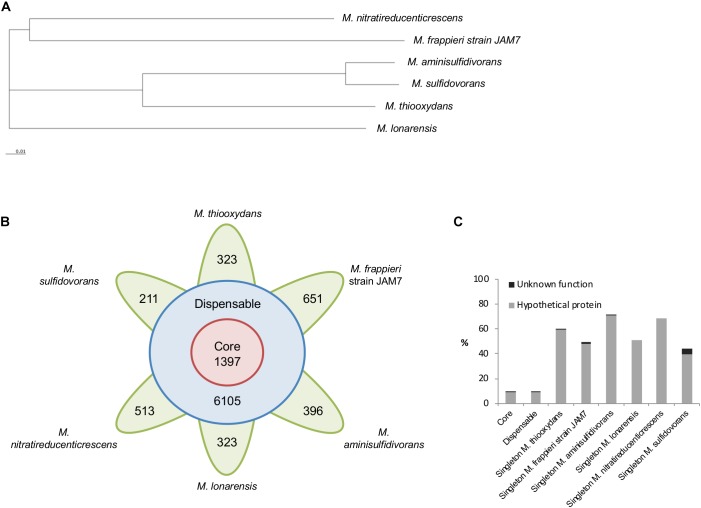
Pan-genome analysis of *Methylophaga* species. **(A)** Pan-genome tree consisting of six *Methylophaga* species was constructed using the neighbor-joining method within the EDGAR platform. **(B)** Number of core, dispensable, and specific genes (singletons) of each *Methylophaga* species. **(C)** Proportion of hypothetical and uncharacterized proteins in the core, dispensable and singleton genome of six *Methylophaga* species.

A comparative genome analysis was carried out to assess to what extent genes of DMS metabolism detected in *M. thiooxydans* are conserved in other members of the genus *Methylophaga*, which differ in their reported abilities to degrade methylated sulfur compounds. At the time of the analysis, genomes for six *Methylophaga* genomes obtained from different environments were chosen (Supplementary Table S7), including three for which growth on DMS has been reported previously (*M. thiooxydans*, *M. sulfidovorans*, *M. aminisulfidivorans*), one which was shown not to grow on DMS (*M. lonarensis*) and two for which DMS degradation has not been tested (*M. frappieri* and *M. nitratireducenticrescens* strain JAM1). Genome sizes range from ∼2.64 to ∼3.26 Mb with GC contents between 40 and 50% (Supplementary Table S1. Analysis of sequence annotations revealed that on average 91% of the genomes consist of coding sequences. Pan-genome analysis, carried out using EDGAR (Blom et al., 2016), identified metabolic genes present in all *Methylophaga* species (core genes), in two or more *Methylophaga* species (accessory or dispensable genes), and ‘unique’ *Methylophaga* species (singleton genes). A pan-genome tree was constructed (Figure 3A) based on the pan-genome dataset and neighbor-joining method (Saitou and Nei, 1987). The *Methylophaga* species exhibiting the least amount of evolutionary change from a common ancestor are *M. aminisulfidivorans* and *M. sulfidovorans* (Figure 3A), these two species also have a higher average amino acid identity score compared to the other four *Methylophaga* species (Supplementary Figure S7). Overall, pan-genome analysis of the six strains identified a total of 11,316 genes, consisting of 1,397 core genes, 6105 dispensable genes and 323, 651, 396, 323, 513, and 211 singletons for *M. thiooxydans*, *M. frappieri* strain JAM7, *M. aminisulfidivorans*, *M. lonarensis, M. nitratireducenticrescens* and *M. sulfidovorans*, respectively (Figure 3B). On average 56.1% of singletons were identified as having hypothetical functions (Figure 3C). The number of singletons did not correlate with the size of the genome, which contrasts with the correlation of the number of genes and the size of the genome.

Investigation of DMS utilization pathways and subsequent C1 oxidation pathways in six *Methylophaga* species revealed the presence of the gene encoding for the methanethiol oxidase involved in degradation of the metabolic intermediate MT in the genomes of *M. thiooxydans* and *M. sulfidovorans* while this gene was not detected in *M. frappieri* strain JAM7, *M. aminisulfidivorans*, *M. lonarensis*, and *M. nitratireducenticrescens* (Supplementary Table S8). None of these *Methylophaga* species contain other known genes of DMS metabolism, including homologs of the DMS monooxygenase (*dmoA*) (Boden et al., 2011a) or the DMS dehydrogenase (McDevitt et al., 2002).

**FIGURE 4:**
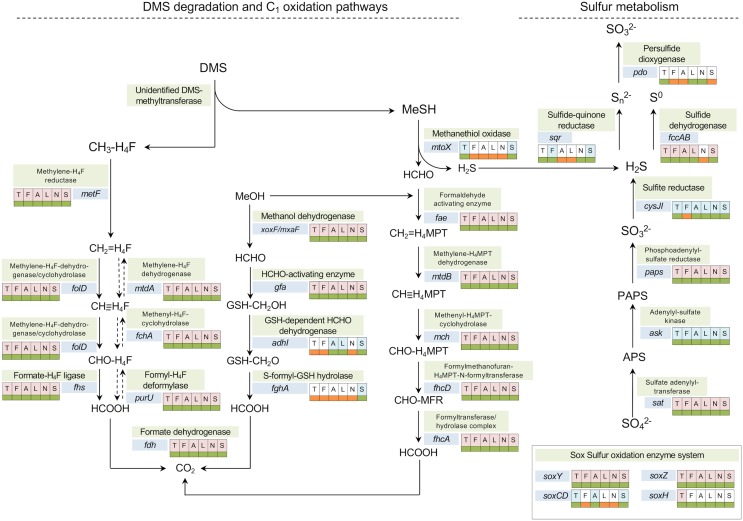
Metabolic pathways involved in DMS degradation/transformation, one-carbon and sulfur oxidation annotated with presence/absence of specific genes in the genomes of *Methylophaga*. The analysis was based on a six-way comparison among *M. thiooxydans* (T), *M. frappieri* (F), *M. aminisulfidivorans* (A), *M. lonarensis* (L), *M. nitratireducenticrescens* (N), and *M. sulfidovorans* (S). The shadings behind the letters indicate presence in core (light red) or dispensable (light blue) genome. The color-coded boxes next to the genes indicate the presence (green) or absence (orange) of a gene in each genome.

Regarding methanol metabolism, all six *Methylophaga* genomes have genes encoding the lanthanide-dependent methanol dehydrogenase XoxF (Hibi et al., 2011; Nakagawa et al., 2012; Pol et al., 2014) of the clade XoxF5 (Keltjens et al., 2014) and the *mxaF* gene, encoding the alpha subunit of the Calcium-dependent methanol dehydrogenase (Williams et al., 2005), which are responsible for the oxidation of methanol to formaldehyde (Harms et al., 1996; Chistoserdova and Lidstrom, 1997) (Figure 4). The lanthanide-dependent methanol dehydrogenase is thought to be more widespread in bacterial genomes (Lv et al., 2018).

All six *Methylophaga* species have genes encoding enzymes of the tetrahydrofolate (H_4_F) linked pathway (Figure 4). Genes encoding 5,10-methylenetetrahydrofolate reductase (*metF*) and the bifunctional enzyme 5,10-methylene-tetrahydrofolate dehydrogenase/cyclohydrolase (*folD*), were detected in all the *Methylophaga* genomes (Figure 4). The formate-tetrahydrofolate ligase, encoded by the gene *fhs* (Figure 4), can have a role in catabolic and anabolic metabolism, either allowing for ATP synthesis during oxidation of formyl-H4F (CHO-H_4_F) to formate or providing C1 units for assimilatory metabolism through energy dependent conversion of formate to formyl tetrahydrofolate; alternatively the oxidation of formyl-H_4_F to formate can also be facilitated by PurU, the formyl-H_4_F deformylase encoded by the *purU* gene (Studer et al., 2002; Marx et al., 2003; Chen, 2012).

Similarly, genes encoding the formaldehyde activating enzyme (*fae*) and further enzymes of the tetrahydromethanopterin (H_4_MPT)-linked pathway of formaldehyde oxidation are also present in all *Methylophaga* genomes. The formate dehydrogenase (FDH, encoded by gene *fdh*) mediates the last step of the H_4_MPT and H_4_F-linked C_1_ oxidation pathways, the oxidation of formate to CO_2_. The genes encoding FDH are present in all *Methylophaga* genomes. Reduction of formaldehyde can also be mediated by the glutathione-dependent formaldehyde-activating enzyme, encoded by the gene *gfa* (Goenrich et al., 2002), which was detected in all six *Methylophaga* species, however, the gene encoding for the glutathione-dependent formaldehyde dehydrogenase (*adhI*) was only detected in *M. aminisulfidivorans* and *M. lonarensis* and the gene encoding the *S*-formyl-GSH hydrolase (*fghA*) was not detected in any of these *Methylophaga* species (Figure 4).

Thus, as expected, comparative genome analysis shows that all six *Methylophaga* species are capable of generating energy from methanol using either lanthanide-dependent or the calcium-dependent methanol dehydrogenases and have pathways of H_4_F and H_4_MPT-linked formaldehyde oxidation. Regarding sulfur metabolism, two *Methylophaga* isolates *M. thiooxydans* and *M. sulfidovorans* may have a common DMS oxidation pathway involving an as-yet unidentified primary enzyme of DMS metabolism suggested to be a DMS methyltransferase (Boden et al., 2010) and methanethiol oxidase, with subsequent formaldehyde oxidation via the H_4_F-dependent pathway and/or the H_4_MPT-dependent pathways. *Methylophaga aminisulfidivorans*, which was reported to grow on DMS and DMSO (Kim et al., 2007), does not have a methanethiol oxidase (*mtoX*). This strain must either possess an as-yet unidentified pathway for the degradation of DMS (and DMSO) or use an alternative methanethiol oxidase. For the oxidation of potentially DMS or DMSO-derived sulfur (sulfide) *Methylophaga aminisulfidivorans* has a sulfide dehydrogenase (*fccAB*) (Figure 4). Regarding inorganic sulfur metabolism, homologs of genes encoding enzymes of sulfide oxidation were found in all strains, but neither SQR nor sulfide dehydrogenases are present in all strains (Figure 4). All strains have genes for assimilation of sulfate to sulfite, and, with the exception of *M. frappieri* all strains had a sulfite reductase. None of the strains contain a complete *sox* system, but some components encoded by the genes *soxCDYZH* were present in most genomes.

## Discussion

Overall, comparative proteomic and transcriptomic experiments of *M. thiooxydans* grown on either DMS or methanol identified the major pathways involved in *M. thiooxydans* during growth on DMS and methanol.

The comparative proteomics experiment of *M. thiooxydans* grown either on DMS or methanol showed a great coverage of proteins across several relevant metabolic pathways, including the primary enzymes involved in methanol degradation, methanethiol degradation, functions involved in formaldehyde degradation and assimilation (such as the KEGG category one-carbon pool by folate, the RuMP cycle, KEGG category methane metabolism) as well as relevant enzymes from central carbon metabolism. Similarly, the transcriptomics analysis identified transcripts of relevant genes, but additional genes activated in either growth condition were also identified.

### Pathways Induced During Growth on DMS

Both methanethiol oxidases present in the genome of *M. thiooxydans* were highly upregulated during growth on DMS as shown by both proteomics and transcriptomics. The downstream genes encoding a fusion of the SCO1/MauG domains present as single genes in *Hyphomicrobium* sp. VS, which are suggested to be important in maturation of the protein, were also both induced during growth on DMS. In line with MT degradation producing H_2_O_2_, induction of catalase and peroxiredoxin reflected important responses to oxidative stress (Rhee, 2016). Membrane lipids are likely also damaged by H_2_O_2_ which may explain the upregulation of genes that might contribute to maintenance of membrane lipid homeostasis including for instance a fatty acid desaturase (MDMS009_226) which may potentially receive reducing power from the product of MDMS009_61, an NAD(P)H flavin reductase that was highly upregulated during growth on DMS (Supplementary Table S6).

What is missing, as far as primary DMS metabolism is concerned, is the presumed DMS methyltransferase which was suggested to be responsible for the first step of DMS degradation in *M. thiooxydans* (Boden et al., 2010). A candidate gene for this putative ‘DMS-methyltransferase’ had not been identified so far and none was detected here as being induced on DMS by proteomics or transcriptomics. There is a methyltransferase in mto-cluster 1, annotated as a ‘methylated DNA-protein cysteine *S*-methyltransferase.’ Although its transcription can be seen in the RNAseq data, the expression levels are similar during growth on methanol and DMS and the protein is not detected in the proteomics data. It most likely plays a role in DNA repair.

Proteomics data indicated that the enzymes of the H_4_F-linked oxidation pathway were upregulated. Thus a methyl group transfer from DMS to H_4_F with its subsequent H_4_F-linked oxidation to CO_2_ is a possibility, similar to the oxidation of the methyl group from methyl chloride in a H_4_F-linked degradation pathway first described in *Methylobacterium extorquens* CM4 (Vannelli et al., 1999). Cleaving off one methyl group from DMS could result in methanethiol as a reaction product, consistent with observed MT oxidation by the presence and expression of MTO (Schäfer, 2007; Boden et al., 2010). The expression of the H_4_F pathway on DMS and methanol suggests that it does not solely serve as a catabolic route though. An alternative, unrecognized route of primary DMS metabolism may exist, such as a hydrolase or monooxygenase. This issue warrants further work, potentially using ^13^C labeling and metabolomic analysis. Formaldehyde is also channeled into the tetrahydromethanopterin-linked pathway of formaldehyde degradation by formaldehyde activating enzyme and degraded to CO_2_.

Proteomics and transcriptomics data support that formaldehyde produced by MTO is assimilated into biomass via the RuMP cycle. Relevant enzymes were detected by both the proteomics and transcriptomics analyses. However, Boden et al. (2010) previously detected activity of the 2-keto-3-deoxy-6-phosphogluconate (KDPG) aldolase (locus MDMS009_149) but none of fructose-1,6-bisphophate aldolase. Previous analysis of the draft genome sequence of *M. thiooxydans* suggested that a fructose-1,6-bisphophate aldolase was absent (Boden et al., 2011b), but a reanalysis showed that the protein-encoding gene for the fructose-1,6-bisphophate aldolase was present in the *M. thiooxydans* genome (MDMS009_1331). Proteomics and transcriptomics analysis showed that fructose-1,6-bisphophate aldolase was expressed, suggesting that enzyme activities of the RuMP cycle need to be reassessed in *M. thiooxydans*.

#### Sulfide Oxidation

Previous work suggested that sulfide dehydrogenase and sulfide oxygenase activities were present. A sulfide dehydrogenase (flavocytochrome *c* sulfide dehydrogenase) was induced during growth on DMS, which produces elemental sulfur. Transcriptomics demonstrated that a second sulfide-degrading enzyme, the sulfide:quinone reductase (SQR) was also highly expressed in cells grown on DMS (locus MDMS009_1966) but not on methanol, which would produce polysulfide, which could then react through an unknown enzyme or chemically with sulfite to yield thiosulfate (S_2_O_3_^2-^, Figure 2) as previously found in *Thiobacillus thioparus* and *Acidithiobacillus thiooxidans* (Suzuki and Silver, 1966) and suggested for *M. thiooxydans* (Boden et al., 2010). The sulfite for this reaction was suggested to be the product of the sulfide oxygenase whose activity was previously measured in *M. thiooxydans*, with the most likely candidates for this reaction being the induced persulfide oxygenases encoded in the vicinity of MTO genes. Several enzymes/genes could therefore be demonstrated which are in agreement with the previous work on the sulfur oxidation pathway in *Methylophaga* (Boden et al., 2010). The final step of this pathway, the oxidation of thiosulfate to tetrathionate has previously been shown to be carried out in *Allochromatium vinosum* by the TsdA thiosulfate dehydrogenase (Denkmann et al., 2012), in *Shewanella oneidensis* by the octaheme tetrathionate reductase (Atkinson et al., 2007) or DoxDA *Acidianus* or *Acidithiobacillus* (Wang et al., 2016), but *M. thiooxydans* does not have homologs of these enzymes. A recent study by Pyne et al. (2018) demonstrated that XoxF PQQ-dependendent dehydrogenases in *Advenella kashmirensis* were capable of oxidizing thiosulfate to tetrathionate. Given the expression of several XoxF enzymes in *M. thiooxydans* both during growth on DMS and methanol, it is possible that the XoxF enzymes may be responsible for the this step of the sulfur oxidation pathway, but this requires experimental confirmation of this activity for any of these enzymes which were previously assumed to have a role in methanol degradation. The expression of some Sox proteins at higher abundance during growth on DMS suggests that these may play a role in oxidation of inorganic sulfur intermediates. Genes encoding SoxA and SoxB were missing, in agreement with the observation that thiosulfate is not oxidized to sulfate in *M. thiooxydans*.

#### Differences in Gene Expression During Growth on Methanol

Highly overexpressed genes in *M. thiooxydans* grown on methanol were those for the Calcium-dependent methanol dehydrogenase and for proteins involved in anthrachelin biosynthesis. Upregulation of the methanol dehydrogenase subunits and the PQQ biosynthesis genes that were induced would be expected. Anthrachelin is a siderophore involved in iron acquisition (Neilands, 1995; Garner et al., 2004). The methanol dehydrogenase is a PQQ-dependent methanol dehydrogenase, and the PQQ accepts the electrons produced during methanol oxidation and passes them to cytochrome *c*, which is an iron containing hemprotein, which might explain why protein-encoding genes involved in the biosynthesis of the iron acquiring siderophore anthrachelin are up-regulated during growth on methanol.

#### Hypothetical Genes

A number of previously unannotated and/or not even predicted genes (Supplementary Table S9) were identified as being expressed. A total of 936 protein-coding transcripts were detected for which no function could be predicted, which accounts for about 30% of all detected protein-coding transcripts. About 20 to 40% of hypothetical proteins are normal in a sequenced genome (Galperin, 2001). For some genes annotated as hypothetical proteins their expression during growth on DMS or methanol associates them with as-yet uncharacterized roles during growth on specific carbon sources and so a first potential association with a metabolic process has been achieved. Further work on these genes will be critical to better understand the specific roles these genes play for growth on DMS or methanol by *Methylophaga*. It reemphasises the need to establish a genetic system for studying gene function in *M. thiooxydans*. Some transcripts were identified as previously unannotated genes. None of these had a predicted function (Supplementary Table S9), but some were highly expressed.

Based on molecular genetics a strong correlation between mRNA expression levels and protein abundance would be assumed (Zhang et al., 2010). However, several studies have failed to demonstrate a high correlation between protein and mRNA abundances (Greenbaum et al., 2002; Nie et al., 2007; Taniguchi et al., 2010; Vogel and Marcotte, 2012). This contradiction underlines the benefit of using proteomics and transcriptomics in combination as application of just one of these approaches is likely to be less representative of the biological system under investigation (Park et al., 2005; Zhang et al., 2010). How well-proteomics and transcriptomics results matched one another is shown in Supplementary Figure S6, which included all transcriptomic and proteomic expression levels. In this study, some proteins were found in higher abundance in *M. thiooxydans* grown on DMS and their corresponding protein encoding genes were often also up-regulated in the transcriptomic experiment (Supplementary Figure S6), e.g., the methanethiol oxidase (MtoX). However, that was only the case for a few proteins. There were also cases where results from proteomic and transcriptomics were somewhat contradictory, e.g., the observation of higher protein levels of a sulfate permease on DMS and higher transcript levels of the same protein on methanol.

## Conclusion

A combined approach of proteomics and transcriptomics analysis has provided more detailed information on pathways involved in DMS and methanol degradation in *M. thiooxydans*, confirming some previous observations made by enzyme assays, and in some cases identifying the genes and proteins responsible for specific activities. At the same time, the data also identify gaps of understanding and identify specific issues that require further investigation, such as the primary mechanism of DMS degradation and the potential role in thiosulfate oxidation of the lanthanide-dependent XoxF methanol dehydrogenases during growth on DMS. Pangenomic analysis showed, as expected for a genus of methylotrophic bacteria, that relevant central pathways such as H_4_F and H_4_MPT-linked formaldehyde and methanol degradation are part of the core genome, but only two of the six investigated *Methylophaga* genomes (*M. thiooxydans* and *M. sulfidovorans*) indicated metabolic potential to utilize methanethiol, the intermediate of DMS degradation, based on the previously identified methanethiol oxidase. This is surprising given the reported ability of *M. aminisulfidivorans* to grow on DMSO and DMS, as well as the close relatedness between *M. sulfidovorans* and *M. aminisulfidivorans* based on the relatively smallest evolutionary change. Overall, these results demonstrate that our understanding of the underlying traits for utilization of methylated sulfur compounds in members of this genus are still inadequate and that more work is required to characterize these metabolic functions using a range of genetics, biochemistry and metabolomics approaches.

## Author Contributions

EK and HS conceived the research, analyzed the data, and wrote the manuscript. EK carried out the experimental research.

## Conflict of Interest Statement

The authors declare that the research was conducted in the absence of any commercial or financial relationships that could be construed as a potential conflict of interest.

## Footnotes

1 http://www.proteomexchange.org

2 https://img.jgi.doe.gov/

## Supplementary Material

Figure S1 SDS-PAGE of protein fractions of *Methylophaga thiooxydans* DMS010 grown on methanol or DMS. **(A)** Soluble protein fraction; lanes 1–3 *M. thiooxydans* DMS010 grown on methanol; lanes 4–6 *M. thiooxydans* DMS010 grown on DMS, S – Precision Plus Protein Standard (Bio-Rad Laboratories, Inc.). **(B)** Membrane protein fraction; lanes 1–3 *M. thiooxydans* DMS010 grown on methanol; lanes 4–6 *M. thiooxydans* DMS010 grown on DMS, S – Precision Plus Protein Standard (Bio-Rad Laboratories, Inc.).

Figure S2 Schematic of the two gene clusters encoding methanethiol oxidases in Methylophaga thiooxydans. The locus tags (MDMS009_XXX) are indicated inside the arrows signifying the coding sequences, the annotation is indicated above the CDS as *mtoX*, methanethiol oxidase; *sco1/mauG*, SCO1/cytochrome c peroxidase MauG domain containing protein; *tlpA*, TlpA_like_family putative metallochaperone (conserved domain cd02966); *pdo*, putative persulfide dioxygenase; *sqrB*, sulfide quinone oxidoreductase; *sulP*, sulfate/sulfite transporter; *cysC*, adenylylsulfate kinase; locus tag MDMS009_97 is annotated as a ‘methylated-DNA–protein-cysteine methyltransferase’; *rpoD*, previously unannotated sigma70 factor. Shading indicates a that the CDS has a predicted signal peptide with a probability > 50%, *sqrB* encoded by MDMS009_274 has a signal P likelihood of 12% (SignalP 5.0; Armenteros et al., 2019); signal peptide predictions for MDS009_211 and MDMS009_894 are based on alternative start sites as discussed in the main text. Scale bar indicates 1 kb.

Figure S3 Graphical summary of signal peptide predictions for the methanethiol oxidase encoded by MDMS009_211 contained on scaffold 1104571000268 (extended to positions 256503:257906). **(A)** Predicted amino acid sequence of the gene extended to the alternative upstream start codon. The underlined sequence indicates additional residues at N-terminal end, the blue highlighted sequence is in agreement with an experimentally determined N-terminal sequence obtained from a polypeptide expressed during growth of *M. thiooxydans* on DMS (Schäfer, 2007). The SignalP 5.0 prediction graphical summary and likelihood for signal peptide for the extended MDMS009_211 protein **(B)** and the gene with the original start codon **(C)**.

Figure S4 Graphical summary of signal peptide predictions for a Sco1/SenC/PrrC domain protein predicted to be involved in cytochrome maturation encoded by MDMS009_894 contained on scaffold 1104571000277 (extended to positions 41243:41737). **(A)** Predicted amino acid sequence of the gene extended to an alternative upstream start codon. The underlined sequence indicates additional residues at N-terminal end. The SignalP 5.0 prediction graphical summary and likelihood for signal peptide for the extended MDMS009_894 protein **(B)** and the gene with the original start codon **(C)**.

Figure S5 Electrophoresis and electropherogram plots for evaluation of purity and concentration of mRNA after enrichment from total RNA from *Methylophaga thiooxydans*. RNA extraction and rRNA depletion led to successful mRNA enrichment. **(A)** Electrophoresis plot showing one clear band for mRNA between 25 and 200 nucleotide (nt) length in samples of *M. thiooxydans* grown on either methanol or DMS. **(B–D)** Electropherogram plots showing mRNA of *M. thiooxydans* grown on methanol (replicates 1 to 3, respectively). **(E–G)** Electropherogram plots showing mRNA of *M. thiooxydans* grown on DMS (replicates 1 to 3, respectively). mRNA concentrations are indicated in the plots and ranged from 14.5 to 26.98 pg/μL in *M. thiooxydans* grown on methanol and from 85.7 to 172.0 pg/μL for *M. thiooxydans* grown on DMS.

Figure S6 Circular representation of the *M. thiooxydans* DMS010 chromosome including proteome and transcriptome expression data. The first (innermost) ring shows the expression (log2 ratio) of the proteome on DMS and methanol grown *M. thiooxydans*, followed by the transcriptome expression (log2 ratio) data (second ring). The third and sixth rings represent the COG categories according to Tatusov et al. (2000). The fourth and fifth rings represent the CDS (blue), tRNA (maroon), and rRNA (purple) on the reverse and forward strand, respectively. The locations of several genes are indicated at the outside of the map.

Figure S7 Average nucleotide identity (ANI) across the six different *Methylophaga* species.

Table S1 Genome characteristics of the six *Methylophaga* genomes.

Table S2 List of query protein sequences used for genome comparison.

Table S3 Summary of protein fractions extracted from *Methylophaga thiooxydans* DMS010 grown in triplicates on either methanol or DMS.

Table S4 Extract of proteins detected in *Methylophaga thiooxydans* DMS010 grown on DMS corresponding to Figure 2. Proteins with gray shading are up regulated in *Methylophaga thiooxydans* DMS010 grown on DMS compared to growth on methanol. Dark gray shadings indicate proteins up regulated more than fourfold, light gray shadings indicate proteins up regulated between 1.3- and 4-fold. Selected proteins that were not detected in the proteome but are present in the genome of *Methylophaga thiooxydans* DMS0101 are included for clarity (marked with ‘-’ in the last column Ratio DMS/Methanol).

Table S5 Hypothetical proteins detected as up-regulated in *Methylophaga thiooxydans* DMS010 grown on DMS compared to growth on methanol.

Table S6 Comparison of transcript expression (mRNA) of *Methylophaga thiooxydans* DMS010 grown on methanol or DMS. Rockhopper calculates one expression level for each treatment (DMS/methanol) and each transcript from the triplicate RNA sequencing results as explained in the text above.

Table S7 Characteristics of six *Methylophaga* species.

Table S8 Presence and absence of genes involved in DMS degradation and sulfur metabolism in six *Methylophaga* species.

Table S9 Previously unannotated and/or not predicted genes in *Methylophaga thiooxydans*.
