# Supplementary figures and images for: Identification of Proteins and Genes Expressed by Methylophaga thiooxydans During Growth on Dimethylsulfide and Their Presence in Other Members of the Genus
Source: Front Microbiol. 2019 May 29;10:1132. doi: 10.3389/fmicb.2019.01132 (PMC6548844; doi:10.3389/fmicb.2019.01132)

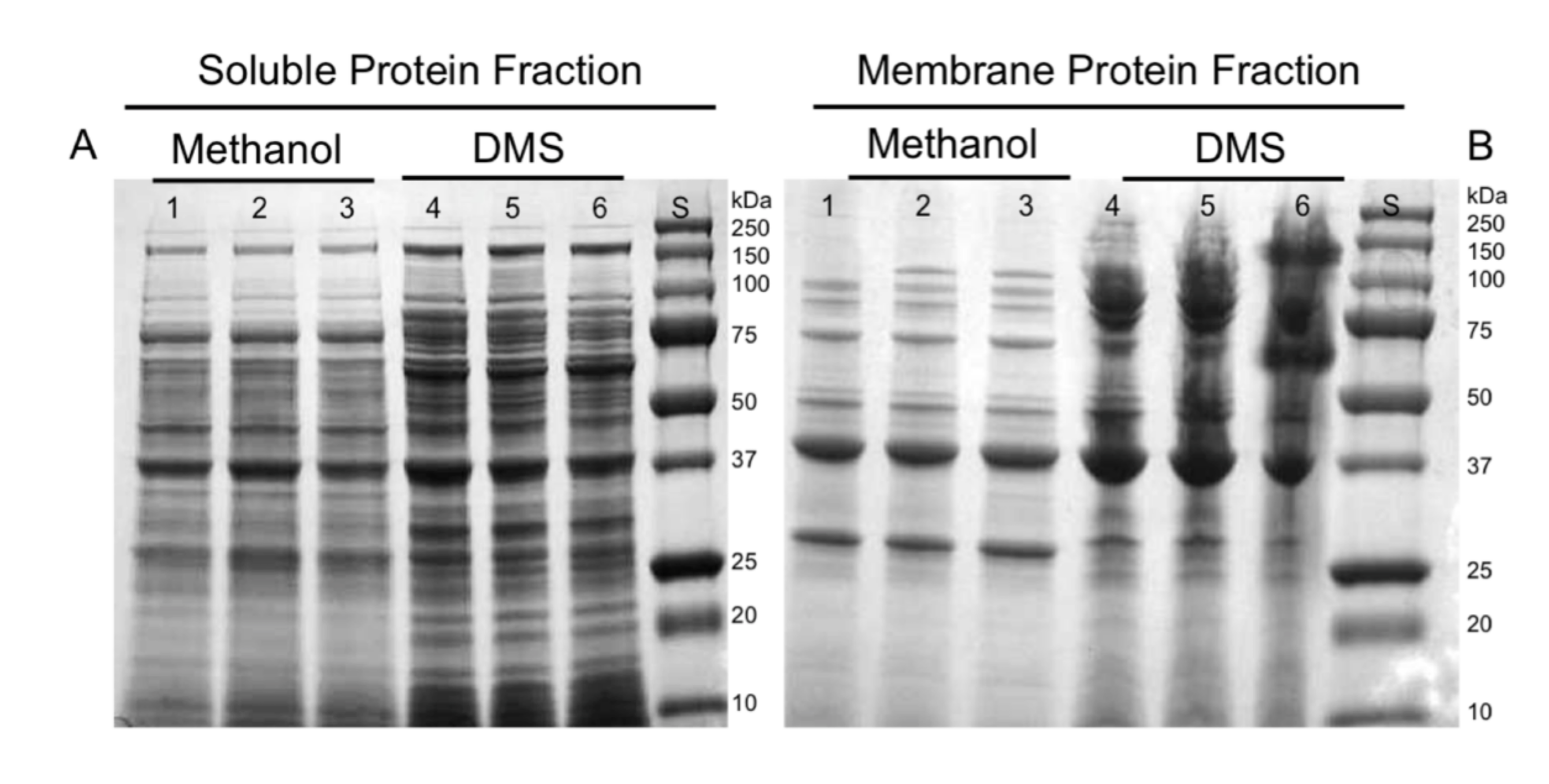

Supplement: Figure S1 — SDS-PAGE of protein fractions of Methylophaga thiooxydans DMS010 grown on methanol or DMS. (A) Soluble protein fraction; lanes 1–3 M. thiooxydans DMS010 grown on methanol; lanes 4–6 M. thiooxydans DMS010 grown on DMS, S – Precision Plus Protein Standard (Bio-Rad Laboratories, Inc.). (B) Membrane protein fraction; lanes 1–3 M. thiooxydans DMS010 grown on methanol; lanes 4–6 M. thiooxydans DMS010 grown on DMS, S – Precision Plus Protein Standard (Bio-Rad Laboratories, Inc.). [file Image_1.TIFF]

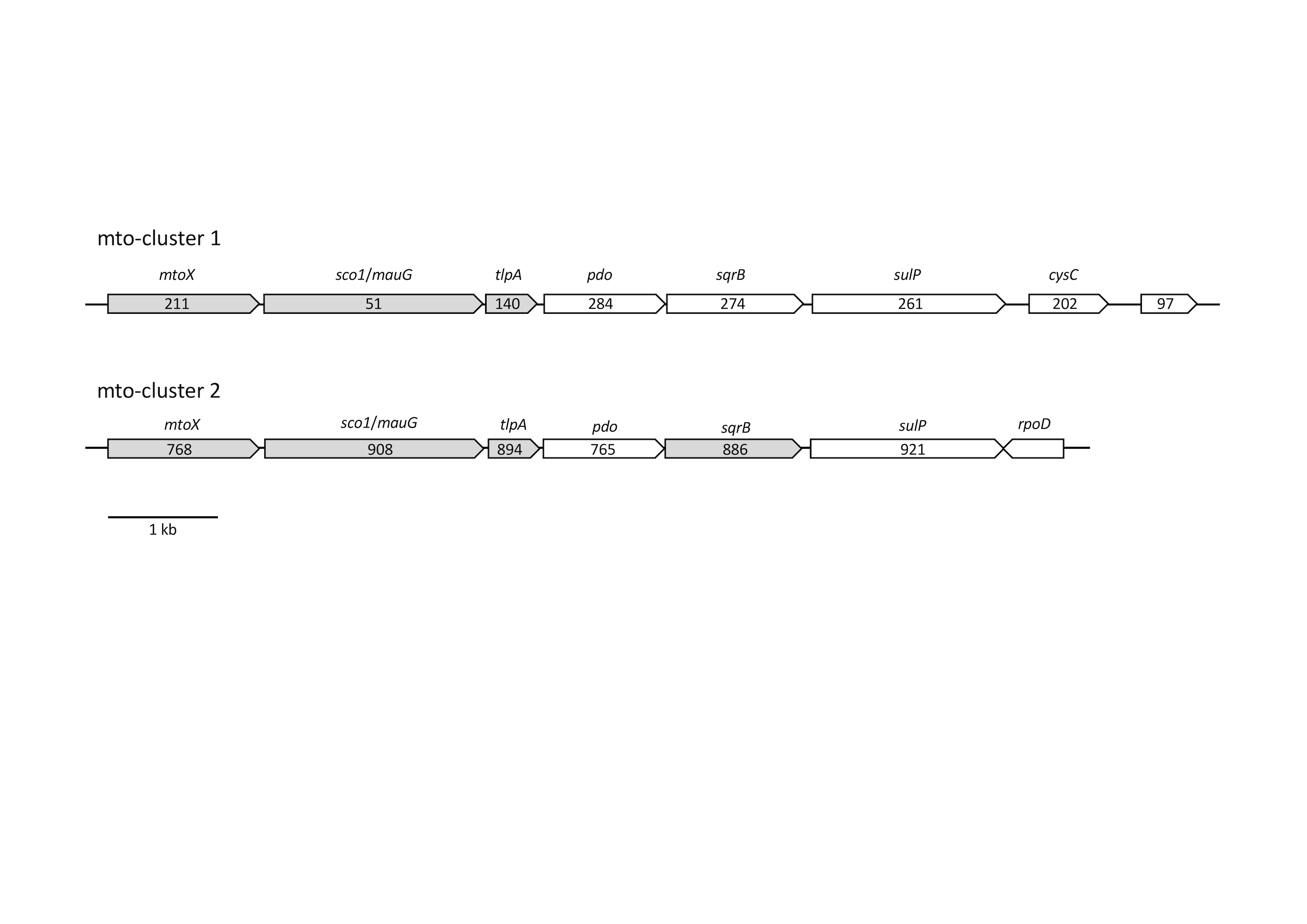

Supplement: Figure S2 — Schematic of the two gene clusters encoding methanethiol oxidases in Methylophaga thiooxydans. The locus tags (MDMS009_XXX) are indicated inside the arrows signifying the coding sequences, the annotation is indicated above the CDS as mtoX, methanethiol oxidase; sco1/mauG, SCO1/cytochrome c peroxidase MauG domain containing protein; tlpA, TlpA_like_family putative metallochaperone (conserved domain cd02966); pdo, putative persulfide dioxygenase; sqrB, sulfide quinone oxidoreductase; sulP, sulfate/sulfite transporter; cysC, adenylylsulfate kinase; locus tag MDMS009_97 is annotated as a ‘methylated-DNA–protein-cysteine methyltransferase’; rpoD, previously unannotated sigma70 factor. Shading indicates a that the CDS has a predicted signal peptide with a probability > 50%, sqrB encoded by MDMS009_274 has a signal P likelihood of 12% (SignalP 5.0; Armenteros et al., 2019); signal peptide predictions for MDS009_211 and MDMS009_894 are based on alternative start sites as discussed in the main text. Scale bar indicates 1 kb. [file Image_2.TIFF]

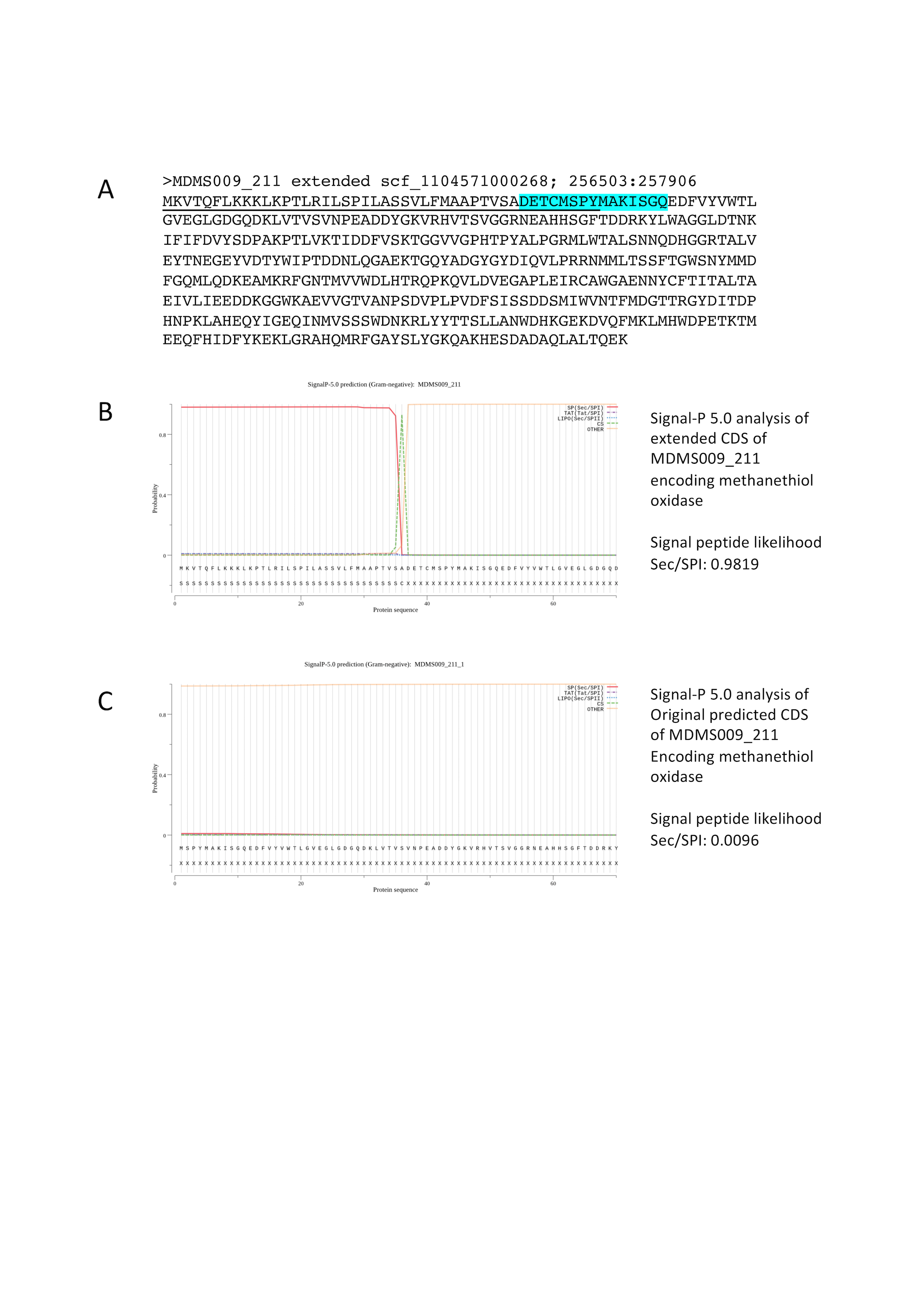

Supplement: Figure S3 — Graphical summary of signal peptide predictions for the methanethiol oxidase encoded by MDMS009_211 contained on scaffold 1104571000268 (extended to positions 256503:257906). (A) Predicted amino acid sequence of the gene extended to the alternative upstream start codon. The underlined sequence indicates additional residues at N-terminal end, the blue highlighted sequence is in agreement with an experimentally determined N-terminal sequence obtained from a polypeptide expressed during growth of M. thiooxydans on DMS (Schäfer, 2007). The SignalP 5.0 prediction graphical summary and likelihood for signal peptide for the extended MDMS009_211 protein (B) and the gene with the original start codon (C). [file Image_3.TIFF]

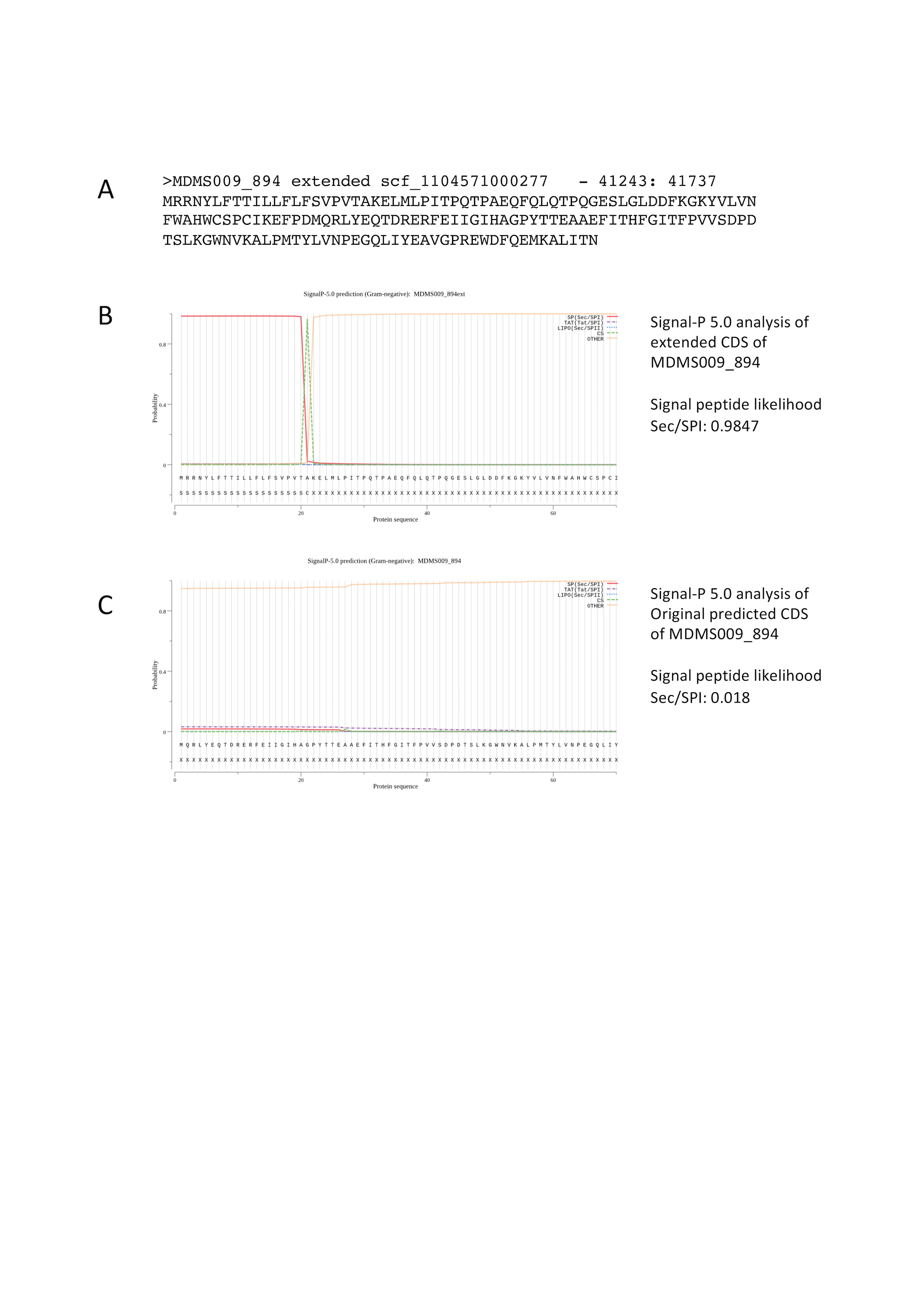

Supplement: Figure S4 — Graphical summary of signal peptide predictions for a Sco1/SenC/PrrC domain protein predicted to be involved in cytochrome maturation encoded by MDMS009_894 contained on scaffold 1104571000277 (extended to positions 41243:41737). (A) Predicted amino acid sequence of the gene extended to an alternative upstream start codon. The underlined sequence indicates additional residues at N-terminal end. The SignalP 5.0 prediction graphical summary and likelihood for signal peptide for the extended MDMS009_894 protein (B) and the gene with the original start codon (C). [file Image_4.TIFF]

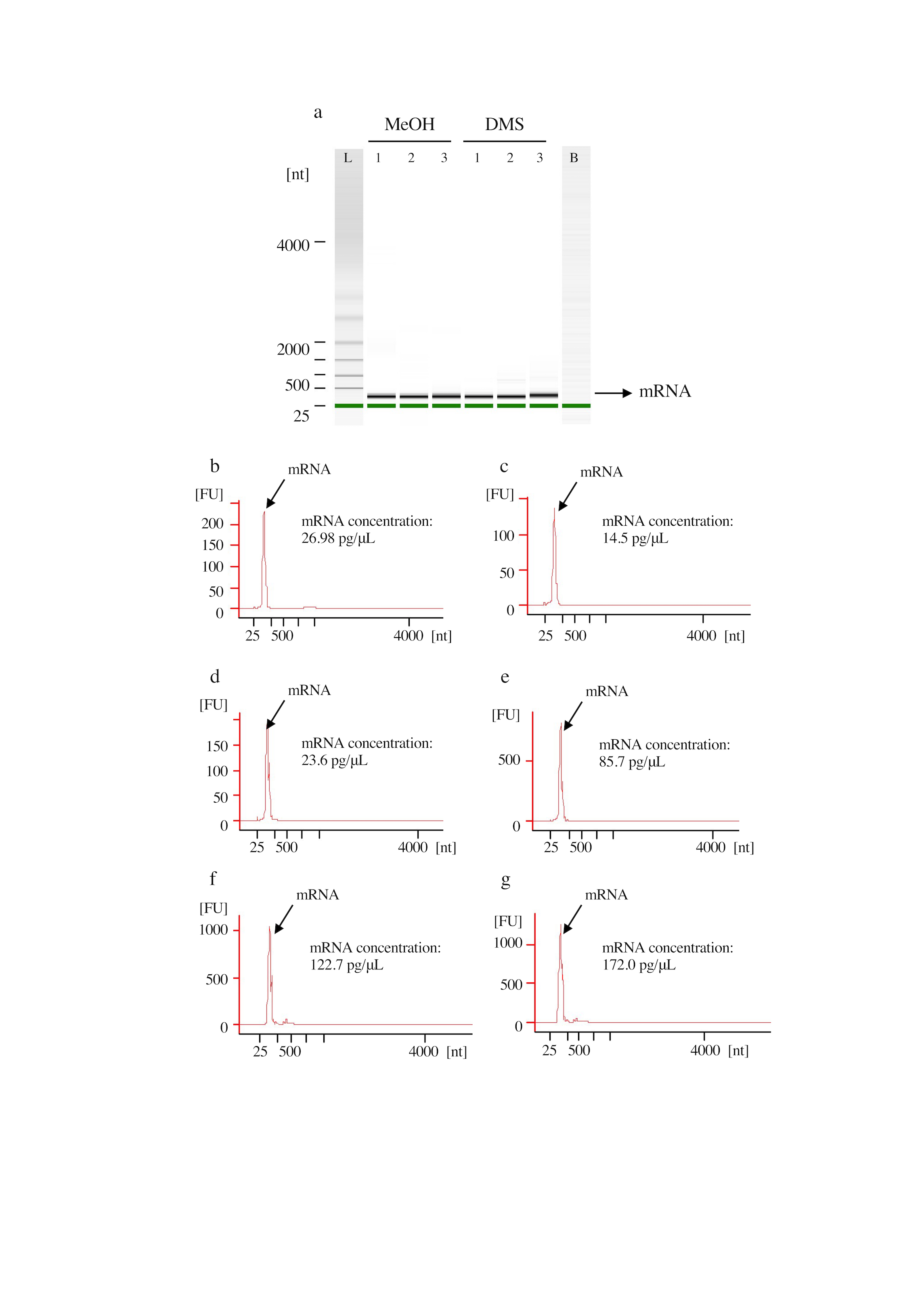

Supplement: Figure S5 — Electrophoresis and electropherogram plots for evaluation of purity and concentration of mRNA after enrichment from total RNA from Methylophaga thiooxydans. RNA extraction and rRNA depletion led to successful mRNA enrichment. (A) Electrophoresis plot showing one clear band for mRNA between 25 and 200 nucleotide (nt) length in samples of M. thiooxydans grown on either methanol or DMS. (B–D) Electropherogram plots showing mRNA of M. thiooxydans grown on methanol (replicates 1 to 3, respectively). (E–G) Electropherogram plots showing mRNA of M. thiooxydans grown on DMS (replicates 1 to 3, respectively). mRNA concentrations are indicated in the plots and ranged from 14.5 to 26.98 pg/μL in M. thiooxydans grown on methanol and from 85.7 to 172.0 pg/μL for M. thiooxydans grown on DMS. [file Image_5.TIFF]

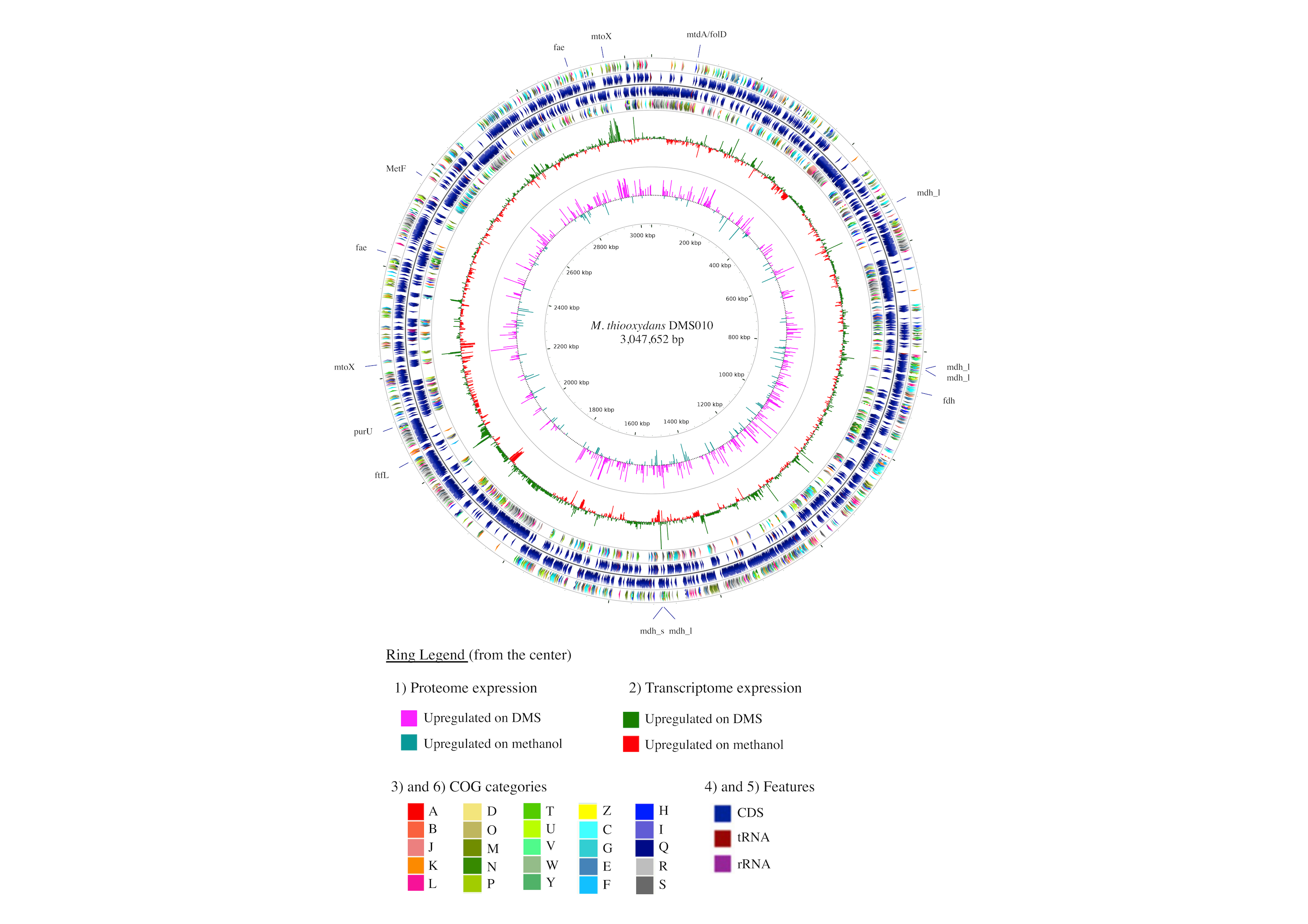

Supplement: Figure S6 — Circular representation of the M. thiooxydans DMS010 chromosome including proteome and transcriptome expression data. The first (innermost) ring shows the expression (log2 ratio) of the proteome on DMS and methanol grown M. thiooxydans, followed by the transcriptome expression (log2 ratio) data (second ring). The third and sixth rings represent the COG categories according to Tatusov et al. (2000). The fourth and fifth rings represent the CDS (blue), tRNA (maroon), and rRNA (purple) on the reverse and forward strand, respectively. The locations of several genes are indicated at the outside of the map. [file Image_6.TIFF]

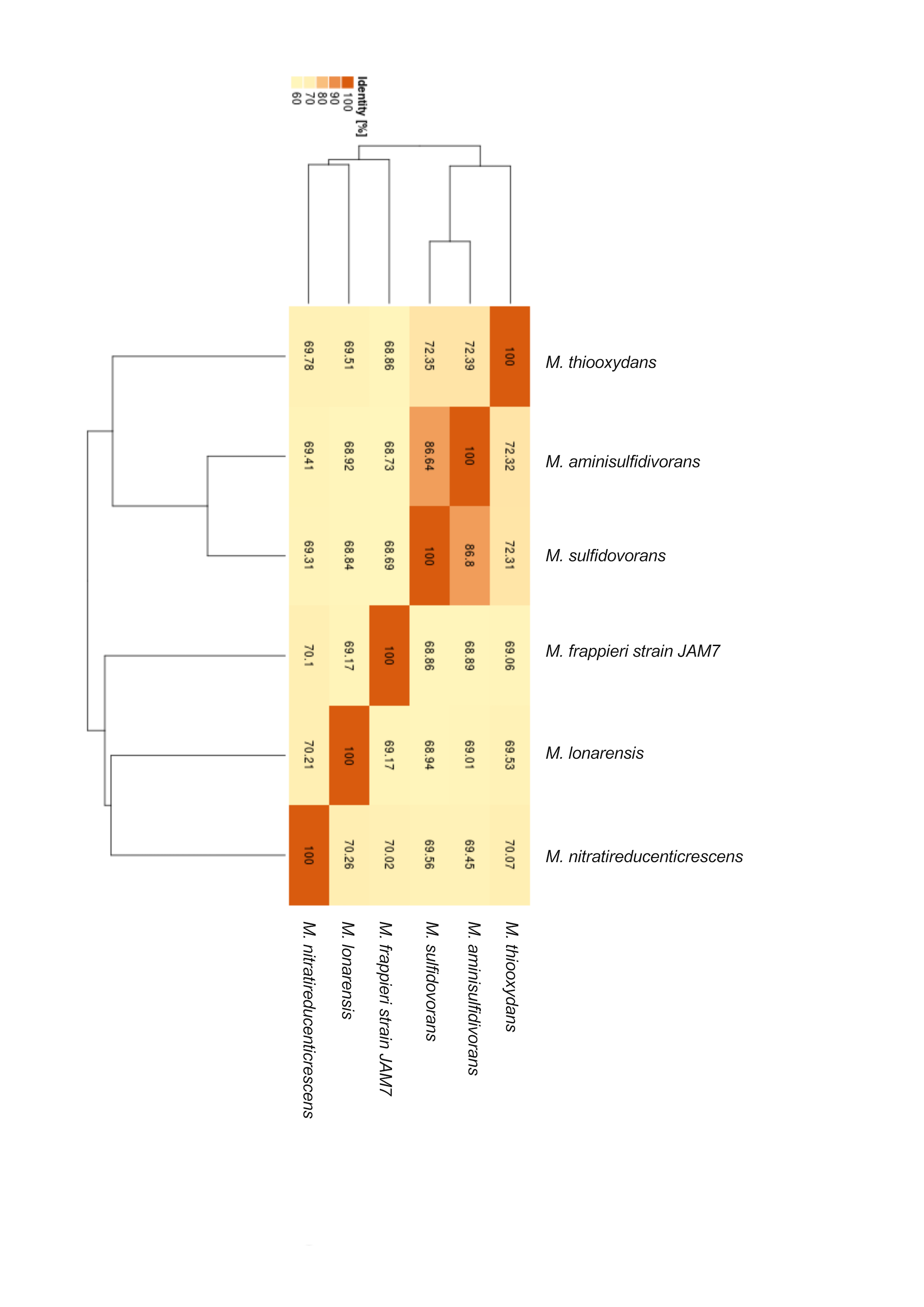

Supplement: Figure S7 — Average nucleotide identity (ANI) across the six different Methylophaga species. [file Image_7.TIFF]
